# An innovative case management intervention for people at high risk of permanent work disability to improve rehabilitation coverage and coordination of health services: a randomized controlled trial (AktiFAME, DRKS00024648)

**DOI:** 10.1186/s12913-022-07482-9

**Published:** 2022-03-15

**Authors:** Lea Remus, Marei Grope, Stella Lemke, Matthias Bethge

**Affiliations:** University of Lübeck, Institute of Social Medicine and Epidemiology, Ratzeburger Allee 160, 23562 Lübeck, Germany

**Keywords:** Case management, Rehabilitation, Disability pension, Randomized controlled trial, Return-to-work, Employment

## Abstract

**Background:**

German social law offers services from various providers and agencies for people with chronic disease or disability. Claiming services requires a high level of information and communication. Dealing with a multitude of actors, such as pension insurance agencies, job centers, employers, physicians, and psychotherapists, and coordinating between and with these actors can delay or prevent the utilization of necessary measures like medical or vocational rehabilitation. We developed a case management intervention for people at high risk of receiving a disability pension to address these challenges.

**Methods/design:**

Our randomized controlled trial tests the effectiveness of the multi-component AktiFAME strategy (Active access, counseling, and case management for people at high risk of permanent work disability). This strategy is comprised of four modules: screening and postal contact, telephone counseling, an initial one-on-one interview, and case management. The randomized controlled trial is complemented by an observational study that exclusively involves the individuals participating in case management and accompanies the implementation of the case management intervention. We enroll 9000 individuals aged 18 to 65 years who were identified as being at increased risk for receiving a disability pension based on administrative data from the German Pension Insurance North but who have not yet applied for rehabilitation. Half of the individuals are randomly assigned to the intervention group and the other half to the control group. The intervention group is contacted and informed about a case management intervention, whereas the control group is not. The primary outcome of the randomized controlled trial is the utilization of rehabilitation services from the German Pension Insurance North (medical or vocational rehabilitation). Data on rehabilitation will be provided by the German Pension Insurance North 1 year after random allocation. Secondary and tertiary outcomes cover additional administrative data (e.g., employment and welfare benefits) and a range of patient-reported outcome measures.

**Discussion:**

This randomized controlled trial is designed to determine the effectiveness of a newly implemented multi-component strategy to promote participation and rehabilitation coverage among individuals at high risk of receiving a disability pension.

**Trial registration:**

German Clinical Trials Register (DRKS00024648, April 27, 2021).

**Supplementary Information:**

The online version contains supplementary material available at 10.1186/s12913-022-07482-9.

## Background

Employment is a key resource for participation in society [1–3]. It ensures income and material security, supports an independent lifestyle, and reduces the risk of poverty in old age by building up pension entitlements. Employment enables social contacts and experiences of self-efficacy, and it can give a sense of purpose and contribution [4]. If people become chronically ill (e.g., due to mental illness), this may jeopardize staying at work and future work participation. In case of long-term and presumably permanent work disability, a disability pension can be granted to reduce income losses. Disability pensions are usually granted for a limited period. After 9 years, they are converted into permanent pensions. Currently, more than 1.8 million people in Germany are receiving a disability pension. Every year, about 350,000 applications for a disability pension are received. Slightly more than half of the completed cases are approved. Despite the high absolute number of disability pensions in Germany, the average individual risk of receiving a disability pension is low: four to five out of every 1000 actively insured persons (i.e., persons who are entitled to a pension on the basis of previous employment and associated social security contributions) start a disability pension each year [5]. The 5-year incidence is just under 2% [6]. Expenditure on disability pensions annually is about 21 billion euros. In 2020, 175,808 people were registered as starting to receive a disability pension, of whom 84,640 were men and 91,168 were women. The average age of retirement was around 53 (i.e., about 14 years before the standard retirement age) [7]. The most important health problem for the granting of a disability pension is mental illness, accounting for about 42% of all new disability pensions, followed by cancer and musculoskeletal disorders [5].

According to Article 27 of the Convention on the Rights of Persons with Disabilities [2], persons with disabilities have “the right to the opportunity to gain a living by work freely chosen or accepted in a labor market and work environment that is open, inclusive and accessible to persons with disabilities”. Earning a living through employment requires assistance in the transition to the general labor market and the promotion of job re-entry for people with disabilities. The range of supporting services provided for this purpose under German social law is broad (e.g., medical rehabilitation, retraining, or job adjustments). Frequently, several of these services are needed to enable people to stay at work or return to work. Different service providers may be involved, such as pension insurance, job centers, the employment agency, social welfare institutions, and health insurance agencies. Employers, occupational health physicians, general practitioners, and psychotherapists are also important players. Moreover, it is often not clear to whom a person has to turn in order to apply for services. To meet these challenges, coordinating and accompanying interventions have been proposed and implemented in recent years [8]. In a systematic review of the effects of coordinating return-to-work interventions, 14 randomized controlled trials with 12,568 employees were summarized [9]. The people included in these studies had musculoskeletal or mental health problems and were on sick leave for at least 4 weeks. The authors of that review did not find any benefits for returning to work in favor of the interventions considered. Although small effects were reported for patient-reported outcomes (e.g., pain and functional capacity), the differences were not clinically significant. The quality of the evidence was considered low due to the risk of bias and the low precision of the effect estimators. The transferability of these results to the support of individuals at high risk of permanent exit from work is, however, severely limited as the included populations had rather low levels of sickness absence (i.e., at least 4 weeks of sickness absence), and the interventions were very heterogeneous. A recent randomized controlled trial from Denmark, accompanied by a very rigorous process evaluation, demonstrated higher return-to-work rates in favor of the intervention group when reaching people that had an obvious need for coordinated support [10].

In our study, we take the return-to-work coordination programs described above and develop them into a multi-component strategy. Our strategy is comprised of four modules: screening of administrative data and postal contact, telephone counseling, an initial one-on-one interview, and a case management intervention. We expect this complex intervention to provide people at high risk of receiving a disability pension with the tailored support they need to sustain their work ability and work participation.

### Objectives

We designed a randomized controlled trial to clarify if our strategy increases the likelihood to apply for rehabilitation services and improves several secondary and tertiary outcomes compared to the usual approach, where people with chronic diseases and disabilities have to coordinate their services themselves. An observational study was nested to assess how well the case management intervention is implemented.

### Trial design

Our study is a randomized controlled trial with two parallel groups with a nested observation study. For our randomized controlled trial, we use an approach proposed by Zelen [11]. After identifying 9000 persons with an increased risk of receiving a disability pension, these individuals are randomly assigned to an intervention group or a control group. Only the intervention group is informed about the possibility of our case management intervention. The superiority of our multi-component strategy will be tested primarily with administratively available data provided by the German Pension Insurance North. These data constitute our primary and secondary outcomes. This allows a pragmatic population-based randomized controlled trial. A similar approach was adopted in the evaluation of other population-based screening programs [12, 13]. Our comparator reflects the current practice whereby people with chronic conditions must first apply for rehabilitation but are not consistently and comprehensively informed in advance by the pension insurance about the options to support their participation. In addition, all individuals will be contacted by the German Pension Insurance North 1 year after random assignment, informed about the study and asked to participate in a postal survey to assess several tertiary outcomes. Moreover, the implementation of the case management is accompanied by an observational study that exclusively includes the individuals participating in the case management intervention.

## Methods

### Study setting

Our intervention is implemented in 206 postal code districts in the German states of Schleswig-Holstein and Mecklenburg-Vorpommern and overseen by seven regional case managers. Outreach contacts usually take place in the home environment of the participants.

### Eligibility criteria

We include persons aged 18 to 65 years who received sickness benefits in the previous year and were at increased risk of receiving a disability pension as indicated by a standardized and validated risk index [6]. All included persons have received at least 180 days of social security contributions from employment or unemployment benefits or sickness benefits in the previous year.

We exclude individuals who have used rehabilitation services in the past 3 years or already receive a disability pension. Some people may be identified as having a high risk of permanent work disability although our case management intervention is not appropriate, e.g., if people require medical care due to an acute illness or need support due to an addiction disorder. We will inform these people about alternative services.

### Treatment

#### Intervention

The multi-component strategy we have developed is comprised of four modules: screening and postal information, telephone counseling, an initial one-on-one interview, and case management. There are not any restrictions on concomitant care and interventions during the trial. Table 1 describes the four components of the multi-component strategy in line with the Template for Intervention Description and Replication (TIDieR) checklist [14].

**Table 1:** Description of the four components according to the TIDieR checklist

Brief name	Postal information	Telephone counseling	Initial one-on-one interview	Case management
Why	Individuals are addressed personally with postal information. The individuals to be contacted are selected by means of a standardized and validated risk index [14]. This score enables sensitive and specific identification of persons who will receive a disability pension without additional intervention. The postal contact is intended to ensure that the selected individuals decide to call the case manager and participate in our case management intervention. In formulating the cover letter, we drew on the self-determination theory [15]. This theory assumes that individuals perform self-determined actions to satisfy their needs for competence, social inclusion, and autonomy. The persons contacted should feel competent to grasp the information through appropriate language. Their autonomy is confirmed, in that the offer is designated as a voluntary measure. The demand for social inclusion is met by the objective of equal participation that we have formulated. The better the cover letter can meet the three criteria, the more the individuals will be motivated to call the case managers. The cover letter also communicates that the three needs can be met as part of the intervention.	The telephone counseling session is intended to clarify all important questions regarding case management, data protection, confidentiality, and voluntariness. It should also pave the way for an initial interview. At the end of the initial telephone contact, the information provided should enable the person to decide whether case management is an option for him/her.	The person and the case manager get to know each other. During the initial interview, all further questions about case management, study participation, data protection, confidentiality and the voluntary nature of participation are clarified. The person should make an informed decision about participating in the case management intervention. The case manager should use the health, personal, and biographical information provided by the participant to develop initial ideas for possible services and support. Finally, the case manager needs to clarify the eligibility of the individual person for our model assessing some medical data.	Case management is a person-centered individual case intervention. We chose this approach to individually assess the multiple interactions of health problem, environment and person described in the model of the International Classification of Functioning, Disability, and Health [16] and to allow shared planning on how to improve the individual’s participation. The involvement of other stakeholders in the social environment recognizes that disability is also a social situation. To increase the likelihood that participants will achieve their goals, goals are formulated as specific, measurable, achievable, relevant, and time-bound. Coaching approaches are used to enhance the participant’s self-reflection skills, acceptance of illness, resilience, and motivation. A resource-based approach aims to activate available skills.
What (materials)	Personalized letters	Guideline for initial telephone contact and computerized documentation	Guideline for initial one-on-one interview; computerized documentation; questionnaire for the collection of medical data to check eligibility; study information; informed consent form (in duplicate); baseline questionnaire	Worksheet “Analysis of needs and agreement on objectives”; computerized documentation; toolbox with working materials on medical and vocational rehabilitation, rehabilitation aftercare, reintegration and job retention (graded return-to-work, company integration management, rights and obligations of the employer, degree of disability, and termination), reorientation and job acquisition (career orientation, job application training), health and therapeutic services, social security and support; follow-up questionnaire at the end of the case management intervention
What (procedures)	The selection of the persons to be contacted is made using a computerized algorithm that combines various characteristics stored in the individual accounts at the pension insurance institution (e.g., age and duration of receipt of sickness benefits and unemployment benefits) [6]. We applied weights when combining the various administrative data. Individuals with a risk index score of at least 60 points and who have received sickness benefits in the past year are contacted by the German Pension Insurance North.	The person contacted calls the case manager using the contact details provided in the personalized letter and is informed in a telephone counseling about the goals and implementation of the case management intervention. An initial one-on-one interview is arranged with the case manager.	The person meets with the case manager at an agreed upon location for an initial one-on-one interview.	Case management includes an initial phase of up to three appointments, a phase of individually tailored support and a final evaluation meeting. Intermediate assessment meetings are held at regular intervals to review and, if necessary, update needs and goals. Individualized assistance includes, among other things, consultations on health and social benefits, joint workplace meeting, support in applying for further services, acquisition of potential new employers or internships, coaching on reorientation and job applications, and educational and psychological interventions.
Who provided	German Pension Insurance North (provision of data and dispatch of information letters) and researchers (calculation of the risk index)	Case manager	Case manager	Case manager
How	Postal and individually	By phone and individually	In presence and individually	In presence and individually; if necessary, with the involvement of other stakeholders (e.g., employer, employee representatives, and company physician)
Where	German Pension Insurance North (provision of data and dispatch of information letters) and University of Lübeck (calculation of the risk index)	Workplace of the case manager	Residence of the person or agreed meeting place	Residence of the participant or agreed meeting place or workplace of the case manager; if necessary, other institutions (e.g., offices and counseling centers)
When and how much	Once per individual in several waves	Once after receiving the postal information and 10 to 15 min	Once after telephone counseling and 1 h	Up to 50 h within 1 year after initial interview and approval by the German Pension Insurance North; familiarization (two to three appointments); monitoring and interim review (every 3 months); final interview (one appointment)
Tailoring	Not planned	Not planned	Not planned	The elements individually tailored to the needs of the participants are derived from the jointly developed goals. The dose of case management is also individual. The upper limit is 50 h. Mandatory and regular interim meetings are held to structure the intervention. The toolbox outlined above, with its various working materials, supports the individual design of the case management intervention.
How well	The calculation of the risk index scores uses administrative data with high validity and is performed with a computer syntax. Sampling is described in a guideline to ensure standardized implementation.	The implementation of the telephone counseling is described in a guideline to ensure standardized implementation. The initial telephone contact is documented by the case manager in a standardized manner. To support the accuracy of the implementation, the telephone contacts are discussed with the researchers in bi-weekly video conferences.	The initial one-on-one interview is described in a guideline to ensure standardized implementation. The initial one-on-one interview is documented by the case manager in a standardized manner. To support the accuracy of the implementation, the interviews are discussed with the researchers in bi-weekly video conferences.	All processes that are mandatory in the case management intervention are described in guidelines to ensure standardized implementation (entry into case management, including needs analysis and goal finding, monitoring and final interview). The toolbox provides materials that can be used by the case managers to customize their approach. The case management is documented in a standardized manner to track the accuracy of the implementation of the guideline, as well as the frequency of use of the toolbox elements and contacts with participants and external institutions. Regular video conferences will be held to support accuracy of implementation throughout the process. At the end of the intervention, all case management participants are asked using standardized questionnaires which case management components they received during the intervention.

#### Control

The control group is identified in the same way as the intervention group but will be not informed about the case management intervention.

### Ancillary and post-trial care

Ancillary and post-trial care are not planned. Compensation for harms due to study participation is also not planned.

### Outcomes and other measures

#### Randomized controlled trial

A complete list of all measured constructs, measurement points and expected scaling of the randomized controlled trial can be found in Table 2. Adverse events will not be systematically assessed.

**Table 2:** Measures, assessment, expected scaling, and measurement occasions in the randomized controlled trial and the nested observational study

*Outcome*	*Source and reference*	*Scaling*	*Baseline*	*End of the case management intervention*	*12-month follow-up*
**Primary outcome**					
Utilization of medical or vocational rehabilitation services	Administrative data from the individual pension insurance account	Binary			X
**Secondary outcomes**					
Employment	Administrative data from the individual pension insurance account	Continuous	X		X
Receipt of unemployment benefits	Administrative data from the individual pension insurance account	Continuous	X		X
Receipt of sickness benefits	Administrative data from the individual pension insurance account	Continuous	X		X
Disability pension	Administrative data from the individual pension insurance account	Binary	X		X
**Tertiary outcomes**					
Physician visits	Own development	Continuous	X^1^		X
Psychosocial health	HEALTH-49 [17]	Continuous	X^1^		X
Self-reported prognosis of employability	SPE [18]	Continuous	X^1^		X
Limitations on participation	IMET [19]	Continuous	X^1^		X
Health state	EQ-5D-5L [20]	Continuous	X^1^		X
Perceived stress	PSS-10 [21]	Continuous	X^1^		X
Social support	F-SozU K-6 [22]	Continuous	X^1^		X
Life satisfaction	SOEP [23]	Continuous	X^1^		X
Information about services supporting participation	Own development	Continuous	X^1^	X^2^	X
Work participation	Own development	Binary	X^1^	X^2^	X
Sick leave duration in weeks	Own development	Continuous	X^1^		X
Work ability	WAS [24]	Continuous	X^1^		X
Sociodemographic data	Own development	Nominal/continuous	X^1^	X^2^	X
Dose delivered: telephone counseling	Computerized sheet (own development)	Binary/continuous		X^2^	
Dose delivered: one-on-one interview	Computerized sheet (own development)	Binary/continuous		X^2^	
Dose delivered: case management	Computerized sheet (own development)	Binary/continuous		X^2^	
Initiated services	Own development	Binary		X^2^	
Needs assessment	Own development	Continuous		X^2^	
Relationship with case manager	Own development	Continuous		X^2^	
Competence of case manager	Own development	Continuous		X^2^	
Goal attainment	Own development	Continuous		X^2^	
Content of case management	Own development	Continuous		X^2^	
Rating of case management components	Own development	Continuous		X^2^	

*EQ-5D-5L* 5-level EQ-5D, *F-SozU K-6* 6-item brief form of the Perceived Social Support Questionnaire (Fragebogen zur Sozialen Unterstützung), *HEALTH-49* Hamburg modules for the assessment of general aspects of psychosocial health for therapeutic practice (Hamburger Module zur Erfassung allgemeiner Aspekte psychosozialer Gesundheit für die therapeutische Praxis), *IMET* Index for the measurement of limitations of participation (Index zur Messung von Einschränkungen der Teilhabe), *PSS-10* Perceived Stress Scale, *SOEP* Socio-Economic Panel, *SPE* Self-reported prognosis of employability, *WAS* Work Ability Score

^1^ randomized controlled trial and observational study; ^2^ observational study only

##### Primary outcome

Our primary outcome is the utilization of medical or vocational rehabilitation services 12 months after random assignment. This information is stored in the individual pension insurance account and will be provided by the German Pension Insurance North.

##### Secondary outcomes

Our secondary outcomes are employment, receipt of unemployment benefits, receipt of sickness benefits, and disability pensions 1 year after random assignment. These data are stored in the individual pension insurance account and will be provided by the German Pension Insurance North. Data on employment, unemployment, and sickness benefits will be reported in days per month.

##### Tertiary outcomes

*Physician visits:* The use of primary medical care is captured by asking for visits to general practitioners and other specialists, as well as hospitalizations [25].

*Psychosocial health:* Psychosocial health is measured with three scales of the HEALTH-49 (Hamburger Modul zur Erfassung allgemeiner Aspekte psychosozialer Gesundheit für die therapeutische Praxis) [17]. We address depression (six items [e.g., suffering from a feeling of hopelessness]), phobic anxiety (five items [e.g., suffering from anxiety or fear about leaving the house]), and interactional difficulties (seven items [e.g., suffering from difficulties showing feelings]) in the past 2 weeks. All items are 5-point scaled (0 = not at all, 1 = somewhat, 2 = moderately, 3 = quite a lot, and 4 = very much). The three total scores are the unweighted means of the corresponding items, with higher values indicating higher levels of impairment.

*Self-reported prognosis of employability:* A brief self-reported measure is used to assess the expected impairment of future employment [18]. Feasibility and psychometric properties of the score were tested thoroughly [18, 26]. The total score is based on answers to three questions: Do you believe that you will be able to continue working until you reach retirement age (certainly, rather yes vs. uncertain, and in no case)? Do you see your current state of health as a permanent threat to your general ability to work (no vs. yes)? Are you currently considering applying for a pension (disability pension) (no vs. yes)? The binary categorized answers are summed to give a total score between 0 to 3, with higher scores indicating a higher risk of permanent work disability.

*Limitations on participation*: We use an abbreviated version of the IMET (Index zur Messung von Einschränkungen der Teilhabe) to assess limitations on participation in eight areas of life (e.g., daily tasks and responsibilities, such as work, school, and household chores) that were derived from the International Classification of Functioning, Disability, and Health. The single items are answered on a scale from 0 to 10, with minimum and maximum representing not impaired and completely impaired [19].

*Health state:* To assess the general health of participants, the 5-level EQ-5D is used [20]. Respondents are asked to indicate their health state by answering questions on five different dimensions: mobility, self-care, usual activities, pain/discomfort, and anxiety/depression. Each dimension has five response levels: no problems, mild problems, moderate problems, severe problems, and extreme problems. A combination of numbers represents a health state, with 11,111 reflecting no problems on any of the five dimensions. The 5-digit health state is then transformed to an index value, which reflects how good or bad this health state is according to the preferences of the German general population [27].

*Perceived stress:* The Perceived Stress Scale (PSS-10) is a widely used and well-established self-report scale for measuring perceived stress [21]. Respondents are asked 10 questions about thoughts and feelings that have occurred in the past month (e.g., How often have you felt nervous and stressed in the last month). Participants indicate on a 5-point scale (never, almost never, sometimes, quite often, or very often) how often they had these feelings in the last month. In addition to a total perceived stress score (0 to 40 points), one score is determined for helplessness (0 to 24 points) and one for self-efficacy (0 to 16 points). Higher values indicate more perceived stress.

*Social support:* To evaluate the degree of perceived social support, we use the brief form of the Perceived Social Support Questionnaire (F-SozU K-6, Fragebogen zur Sozialen Unterstützung) [22]. The respondents are asked to rate six statements that assess support from the social environment (e.g., having a very trusted person whose help one can always count on). Statements are rated with a 5-point scale, ranging from 1 (does not apply) to 5 (exactly applicable). The total score is calculated by averaging the single ratings.

*Life satisfaction:* We use eight items of the German Socio-Economic Panel to assess satisfaction in seven life areas (e.g., health or work) and with life itself [23]. Responses are 11-point scaled ranging from 0 (totally dissatisfied) to 10 (completely satisfied).

*Information about services supporting participation:* Four items are used to assess knowledge about services supporting participation (financial support, medical rehabilitation, vocational rehabilitation, and services for participation in society). Participants rate from 0 (strongly disagree) to 5 (strongly agree) whether they are informed about these services.

*Work ability*: Self-reported work ability is assessed using the single-item Work Ability Score, which asks for current work ability compared with lifetime best [24]. The score ranges from 0 (completely unable to work) to 10 (work ability at its best). Higher scores indicate better work ability. The Work Ability Score is highly correlated with the entire Work Ability Index score [28, 29].

*Work participation*: We assess employment state (employed vs. unemployed) and the job title to describe work participation. In addition, we also ask for sickness absence (current state and duration in the past 12 months).

##### Other measures

Age and gender are derived from administrative records. Additional sociodemographic data is assessed by questionnaire and relates to the native language, partnership, children, and educational level.

#### Observational study

*Delivered dose:* As part of the observational study, case managers document the intervention components (i.e., telephone counseling, one-on-one interview, and the case management intervention) in a standardized manner using computerized sheets.

*Received dose:* We use the same items assessing knowledge about services supporting participation as in the randomized controlled trial also in the observational study. In addition, we ask which of these services were initiated. Furthermore, the participants rate five statements about how individual needs and goals were identified, five statements about their relationship with the case manager, three statements about the case manager’s competence, five statements about goal attainment, and nine statements about contents of the case management intervention. Ratings of these statements use a 4-point scale from 0 (do not agree) to 3 (completely agree). Finally, we also ask the participants to rate the different components of our multi-component strategy (e.g., the telephone contact), with grades from 1 (very good) to 5 (insufficient).

### Participant timeline

Table 3 shows the full schedule of enrollment, interventions and assessments.

**Table 3:** Schedule of enrollment, intervention, and assessments

*Timepoint*	*Pre-intervention*	*Beginning of intervention*	*End of intervention*	*12-month follow-up*
**Enrollment**				
Screening and postal information	X			
Randomization	X			
**Interventions**				
Telephone counseling		X		
Initial one-on-one interview		X		
Case management		Continously		
**Assessments**				
Baseline questionnaire		X		
Questionnaire at the end of the case management intervention			X	
12-month follow-up questionnaire				X
Computerized documentation by case managers		Continously		

### Sample size

We will include 9000 persons (intervention group: *n* = 4500; control group: *n* = 4500) in order to detect an increase in claiming a rehabilitation from 10 to 12% in the intervention group (two-sided type I error rate: 5%, power: 80%). We expect that 12.5% of individuals who are informed about our case management intervention will contact our case managers. Of whom, half will join the one-on-one interview, and of whom, 80% will start a case management intervention. This is in line with our pilot study showing that about 5% of the informed individuals participated in a case management intervention. About half of them are anticipated to claim rehabilitation.

### Recruitment

To identify individuals who are at increased risk of receiving a disability pension, the German Pension Insurance North transfers pseudonymized administrative data to the University of Lübeck. In addition to the personal identification number, this data set contains age, gender, and nationality, as well as income and the duration of receipt of short- and long-term unemployment benefits, and sickness benefits for the past 3 years. We summarize these data into a standardized and validated risk index score with a mean of 50 points and a standard deviation of 10 points [6]. High scores indicate an increased risk of receiving a disability pension. Of individuals with a risk index score of at least 60 points, a random sample is drawn. This sampling is done twice: 4500 people are identified in 2021, and another 4500 people will be identified in 2022. In total, 9000 individuals with a high risk index score will be selected for our study. These individuals are randomly assigned equally to the intervention or control group in both years. The samples are transmitted to the German Pension Insurance North who informs the intervention group in several waves about the case management intervention. A total of 225 individuals are targeted for participation in case management and will be accompanied by an observational study.

### Allocation

The principal investigator (MB) generates random numbers using Stata 16.0 to split both random samples of people with a risk index score of at least 60 points (one in 2021 and one in 2022) into the intervention or control group. Allocation is concealed from the case managers, who are not aware of group assignments until participants contact them. Sample selection and random assignment by the principal investigator uses a data set with pseudonyms. This data set is provided by the German Pension Insurance North electronically. After randomization, the principal investigator transmits the pseudonyms and group assignment also electronically to the German Pension Insurance North. Only the pension insurance agency can link pseudonyms to actual individuals to inform them about the intervention, to collect primary and secondary outcomes from administrative records and to contact participants to send them questionnaires to gather tertiary outcomes. Enrollment for the nested observational study is done by the case managers.

### Blinding

Case management intervention participants will be fully aware of their group assignment when we ask them to contact our case managers. Participants in the control group will not learn their group assignment until we ask them to complete the questionnaires to collect tertiary outcomes data. Primary and secondary outcomes data are generated by the data department of the pension insurance agency without knowledge of group assignment. The principal investigator and data analysts will be aware of group assignments when analyzing the data. Unblinding of the control group before assessing tertiary outcomes data is not intended.

### Data collection

Outcomes are collected using administrative data from the German Pension Insurance North, patient questionnaires and computerized documentation by case managers (Tables 2 and 3). The administrative data capture our primary and secondary outcomes of the randomized controlled trial completely, reliably, and validly for both the intervention and control group participants. These data are transmitted electronically, encrypted and pseudonymized by the German Pension Insurance North to the University of Lübeck. The patient questionnaire to assess tertiary outcomes of the randomized controlled trial will be sent out 12 months after random assignment by the German Pension Insurance North with a return envelope addressed to the University of Lübeck. The questionnaires of the observational study are handed out to the participants by the case managers and returned to them completed or handed out together with a return envelope addressed to the University of Lübeck. The computerized case manager documentation was developed with the case managers to ensure the feasibility of the documentation, and all case managers were trained on the required documentation before the randomized controlled trials began. Case managers send their computerized documentation of the telephone counseling, the one-on-one interview, and the case management intervention weekly to the researchers, who check it for completeness and validity. The patient forms and computerized documentation of the case managers were tested in a previous pilot study. If participants withdraw their consent, the collected data will be deleted.

The use of administrative data will ensure complete coverage of primary and secondary outcomes from all study participants. Moreover, we created a website to transparently inform study participants about the study and to maintain interest in the study. Three weeks after the first mailing of the 12-month follow-up questionnaire a single reminder is sent, again containing the questionnaire and the return envelope addressed to the University of Lübeck.

### Data management

A comprehensive data protection concept was developed with the data protection officer of the German Pension Insurance North and clarifies the data processing, rights of participating persons, and technical and organizational measures to ensure the secure and confidential collection, processing, and storage of data. Administrative data are provided by the German Pension Insurance North. Personal data is removed, replaced with the unique study identifier, and submitted to the principal investigator. Questionnaire data are entered, reviewed and exported to statistical software packages for further analysis. Data input and data verification are performed by trained research assistants. The administrative and questionnaire data will be linked using a unique study identifier. Access to the data is limited to the authors and research assistants on the research team, and data management is performed by the authors.

### Statistical methods

We will use Cox regressions to analyze our primary outcome and our time-to-event secondary outcome (i.e., disability pension) and report hazard rate ratios and 95% confidence intervals. Poisson regressions with baseline adjustments will be calculated to estimate incidence rate ratios and 95% confidence intervals in our analysis of our continuous secondary outcomes. We will use linear regressions to analyze our continuous tertiary outcomes. The use of administrative data will ensure that all individuals can be included in our analysis as randomized (intention-to-treat).

Subgroup analyses examine whether estimates differ for the first and second cohorts of participants. The analysis of our nested cohort study of case management participants only will describe the implementation of the case management intervention and changes during the case management intervention. We will use paired t-tests for continuous outcomes and McNemar tests for binary outcomes when analyzing the change between the start and end of the case management intervention.

Analyses of our primary and secondary outcomes will not be affected by non-responses or patient withdrawals due to the use of administrative data, but tertiary outcomes may be missing due to non-response or incomplete response to the 12-month follow-up questionnaire. We will use multiple imputation to augment incomplete responses to the 12-month follow-up questionnaire.

We will not perform interim analyses and did not specify a stopping rule. Statistical test will be regarded as significant if the two-sided p-value of a test is less than 0.05. An up-to-date version of Stata (StataCorp, College Station, Texas, USA) will be used to conduct analyses.

## Discussion

The purpose of our large-scale randomized controlled trial is to test the effects of a new multi-component strategy for individuals with a high risk of receiving a disability pension developed to improve coverage of rehabilitation and the coordination of support for people with chronic diseases and disabilities. We provide updated information on our trial website www.aktifame.de. All results of our study will be published as articles in peer-reviewed journals and at conferences. The authors of this protocol will write the final trial publications. The use of professional writers is not intended. The researchers and the German Pension Insurance North will design a flyer providing information about the key findings of our study (circulation: 2000 copies). These will be sent out nationwide. We will also host a symposium to provide information about our study.

The study protocol was designed using the SPIRIT (Standard Protocol Items: Recommendations for Interventional Trials) checklist [30].

### Trial status

Recruitment has started and is ongoing.

## Supplementary Information

**Additional file 1.** Items from the World Health Organization Trial Registration Data Set

**Additional file 2.** Information on 12-month follow-up for the intervention group of the randomized controlled trial

**Additional file 3.** Information on 12-month follow-up for the control group of the randomized controlled trial

**Additional file 4.** Information on participation in the nested cohort study

**Additional file 5.** Consent form of the nested cohort study

## Abbreviations

AktiFAME: Active access, counseling, and case management for people at high risk of permanent work disability
F-SozU K-6: 6-item brief form of the Perceived Social Support Questionnaire (Fragebogen zur Sozialen Unterstützung)
HEALTH-49: Hamburg modules for the assessment of general aspects of psychosocial health for therapeutic practice (Hamburger Module zur Erfassung allgemeiner Aspekte psychosozialer Gesundheit für die therapeutische Praxis)
IMET: Index for the measurement of limitations of participation (Index zur Messung von Einschränkungen der Teilhabe)
PSS-10: Perceived Stress Scale
SOEP: Socio-Economic Panel
SPE: Self-reported prognosis of employability
SPIRIT: Standard Protocol Items: Recommendations for Interventional Trials
TIDieR: Template for Intervention Description and Replication
WAS: Work Ability Score
