# Supplementary material for: An innovative case management intervention for people at high risk of permanent work disability to improve rehabilitation coverage and coordination of health services: a randomized controlled trial (AktiFAME, DRKS00024648)
Source: BMC Health Serv Res. 2022 Mar 15;22:342. doi: 10.1186/s12913-022-07482-9 (PMC8922787; doi:10.1186/s12913-022-07482-9)
Supplement: Supplementary file 1 — Additional file 1. Items from the World Health Organization Trial Registration Data Set [file 12913_2022_7482_MOESM1_ESM.pdf]

Additional file 1: Items from the World Health Organization Trial Registration Data Set

Data category	Information
Register	German Clinical Trials Register
Last refreshed on	29 November 2021
Main ID	DRKS00024648
Date of registration	27/04/2021
Prospective Registration	Yes
Primary sponsor	Universität zu Lübeck, Institut für Sozialmedizin und Epidemiologie
Public title	Active access, counseling and case management for people at high risk of permanent work disability
Scientific title	Active access, counseling and case management for people at high risk of permanent work disability - AktiFAME
Date of first enrolment	25/06/2021
Target sample size	9000
Recruitment status	Recruiting
URL	http://www.drks.de/DRKS00024648
Study type	Interventional
Allocation	Randomized controlled trial
Masking	Open (masking not used).
Control	Control group receives no treatment.
Assignment	Parallel
Purpose	Treatment

Phase	N/A
Countries of recruitment	Germany
Contacts	Matthias Bethge<br>Ratzeburger Allee 160, 23562 Lübeck, Germany<br>+49 451 50051280<br>matthias.bethge@uksh.de<br>Universität zu Lübeck, Institut für Sozialmedizin und Epidemiologie
Key inclusion and exclusion criteria	Inclusion criteria: We will include persons from 206 postal code districts in Schleswig-Holstein and Mecklenburg-Western Pomerania receiving sickness absence benefit in the previous year and at increased risk of receiving a disability pension—i.e., a risk score of at least 60 points. All included individuals had at least 180 days of social security contributions from employment or unemployment benefits or sickness benefits in the previous year (sum of the three contribution sources).<br>Exclusion criteria: We will exclude individuals who have used rehabilitation services in the past three years, require medical care due to an acute illness, need support due to an addiction disorder, or already receive a disability pension.<br>Age minimum: 18 Years<br>Age maximum: 65 Years<br>Gender: Both, male and female
Health conditions or problems studied	Chronic health problems

Interventions	Intervention 1: The multi-component strategy we have developed comprises four modules: screening and postal information, telephone counseling, initial one-on-one interview, and case management.<br><br>Screening and postal information. We use a standardized and validated risk index that merges various characteristics stored as administrative data at the pension insurance institution (e.g., age, duration of receipt of sickness absence benefits and unemployment benefits). We apply weights when combining the various administrative data. Individuals with a risk index score of at least 60 points and who have received sickness absence benefits in the previous year will be individually mailed once in several rounds by the German Pension Insurance North.<br><br>Telephone counseling. The persons addressed will call the case manager using the contact data provided in the cover letter and are informed once in a 10-minute telephone conversation about the goals and implementation of the case management intervention. A personal initial meeting will be arranged with the case manager.<br>Initial one-on-one interview. The person will meet with the case manager once at an agreed venue for a one-hour initial interview.<br><br>Case management. Case management is a person-centered individual intervention and is carried out by Brücke Schleswig-Holstein gGmbH and Berufsförderungswerk Stralsund GmbH. Up to 50 hours are planned for case management within one year. Case management is structured in an initial phase of up to three appointments, an individually designed support phase, and a final evaluation meeting. Interim assessment meetings are held at regular intervals to review and, if necessary, update needs and goals. The individually designed core phase includes, among other things, consultations on health and social benefits, joint workplace visits, support in applying for further services, acquisition of potential new employers or internships, coaching on reorientation and job applications, and educational and psychological interventions.<br><br>Intervention 2: The control group is identified in the same way as the intervention group but will be not informed about the case management intervention.
Primary outcome	Our primary outcome is the utilization of medical or vocational rehabilitation twelve months after random assignment. This information is stored in the individual pension insurance account and will be provided by the German Pension Insurance North.
Secondary outcomes	Our secondary outcomes are employment, receipt of unemployment benefits, receipt of sickness benefits, and disability pensions one year after random assignment. These data are stored in the individual pension insurance account and will be provided by the German Pension Insurance North.<br><br>Our tertiary outcomes are physician visits (Bethge et al. 2012), anxiety (0 to 4 points; Rabung et al. 2009), depression (0 to 4 points; Rabung et al. 2009), interactional difficulties (0 to 4 points; Rabung et al. 2009), expected impairment of future employment (0 to 3 points; Mittag and Raspe 2003), limitations on participation (0 to 80 points; Deck and Bürger 2005 [modified]), quality of life (0 to 1 points; Herdman et al. 2010), perceived stress (total: 0 to 40 points; helplessness: 0 to 24 points; self-efficacy: 0 to 16 points; Reis et al. 2019), social support (6 to 30 points; Lin et al. 2019), satisfaction in various areas of life (0 to 10 points; Kantar Public 2020), information about services supporting participation (0 to 5 points; own development), self-rated work ability (0 to 10 points; Ilmarinen 2007), and duration of sickness absence in the past twelve

	months (0 to 52 weeks; own development). These data will be collected twelve months after randomized assignment using questionnaires.
Secondary ID(s)	21-073 (Ethics Committee University of Lübeck)<br>U1111-1263-7448 (Universal Trial Number)
Source of monetary support	Knappschaft-Bahn-See - Fachstelle rehapro im Auftrag des Bundesministeriums für Arbeit und Soziales
Status of ethics review	Approved
Approval date of ethics review	19/04/2021
