# Supplementary material for: An innovative case management intervention for people at high risk of permanent work disability to improve rehabilitation coverage and coordination of health services: a randomized controlled trial (AktiFAME, DRKS00024648)
Source: BMC Health Serv Res. 2022 Mar 15;22:342. doi: 10.1186/s12913-022-07482-9 (PMC8922787; doi:10.1186/s12913-022-07482-9)
Supplement: Supplementary file 2 — Additional file 2. Information on 12-month follow-up for the intervention group of the randomized controlled trial [file 12913_2022_7482_MOESM2_ESM.pdf]

German Pension Insurance North, Ziegelstraße 150, 23556 Lübeck

Name
Address

Lübeck, yyyy-mm-dd

Active access, counseling and case management for people at high risk of permanent work disability

Name of case manager
N. N.

German Pension Insurance North
N. N.

INFORMATION

about survey participation for the evaluation of the case management program AktiFAME

Dear Ms. ..., Dear Mr. ...,

About a year ago, you received an invitation from German Pension Insurance North to participate in the new case management program called "AktiFAME." Let me remind you once again: AktiFAME is a novel strategy that we developed together with the University of Lübeck, Brücke Schleswig-Holstein gGmbH and Berufsförderungswerk Stralsund GmbH to improve the participation of people with health impairments. Some of you have taken up this offer, others have not.

Why are we writing to you again today?

To evaluate the effectiveness of our new case management program, we need your support. We can only carry out this evaluation if both the people who have participated in case management and the people who have refrained from participating in case management tell us how they are doing today in terms of their health. We would therefore like to ask you to complete the enclosed questionnaire for us.

Gefördert durch:

Bundesministerium für Arbeit und Soziales

aufgrund eines Beschlusses des Deutschen Bundestages

rehapro

Brücke SH

BFW Berufs-Förderungswerk Stralsund

Deutsche Rentenversicherung Nord

Universität zu Lübeck

What can you do?

Please fill out the enclosed questionnaire independently and completely. Then put it in the enclosed return envelope (fee to be paid by recipient). Then send the envelope directly to the University of Lübeck.

Link with data from your insurance account

By returning the letter, you agree that selected pseudonymized data from your insured person's account can be linked to the information in the questionnaire. This information relates to all rehabilitation measures applied for at the German Pension Insurance North in the last year. In addition, we – German Pension Insurance North – will inform the University of Lübeck whether you are employed.

Voluntariness

We would like to point out once again that completing the questionnaire is voluntary. If you decide not to complete the questionnaire, you do not need to do anything. There will be no disadvantages for you. You can also simply ignore the reminder that you will receive in about three weeks.

Request for your participation

We would be delighted if you would complete the questionnaire. Your information is extremely valuable to us as we aim to improve and develop our services.

Of course, I am at your disposal for any further questions!

With kind regards,

N. N.

German Pension Insurance North

Active access, counseling and case management for people at high risk of permanent work disability

Name of case manager
N. N.

German Pension Insurance North
N. N.

Gefördert durch:

Bundesministerium für Arbeit und Soziales

aufgrund eines Beschlusses des Deutschen Bundestages

rehapro

Brücke SH

BFW Berufsförderungswerk Stralsund

Deutsche Rentenversicherung Nord

Universität zu Lübeck
