# Supplementary material for: An innovative case management intervention for people at high risk of permanent work disability to improve rehabilitation coverage and coordination of health services: a randomized controlled trial (AktiFAME, DRKS00024648)
Source: BMC Health Serv Res. 2022 Mar 15;22:342. doi: 10.1186/s12913-022-07482-9 (PMC8922787; doi:10.1186/s12913-022-07482-9)
Supplement: Supplementary file 3 — Additional file 3. Information on 12-month follow-up for the control group of the randomized controlled trial [file 12913_2022_7482_MOESM3_ESM.pdf]

German Pension Insurance North, Ziegelstraße 150, 23556 Lübeck
*Name*
*Address*

Lübeck, yyyy-mm-dd

**Active access, counseling and case management for people at high risk of permanent work disability**

**Name of case manager**
**N. N.**

**German Pension Insurance North**
**N. N.**

**INFORMATION**

**about survey participation**

Dear Ms. ..., Dear Mr. ...,

About a year ago, together with the University of Lübeck, Brücke Schleswig-Holstein gGmbH and Berufsförderungswerk Stralsund GmbH, we developed a novel case management program to improve the participation of people with health impairments. The new strategy is called "AktiFAME" and is funded by the German Federal Ministry of Labor and Social Affairs as part of the rehapro federal funding program. It is a new supportive service provided by case managers that has not yet been available in the standard care system.

**AktiFAME and our concern**

In order for the new offer to be adopted in standard care, the effectiveness of the offer must be demonstrated in a study. This study is currently being conducted by the University of Lübeck. A year ago, the University of Lübeck started a study in compliance with data protection regulations to determine the probability that insured persons of German Pension Insurance North will leave the workforce before the regular retirement age for health reasons. For this purpose, the University of Lübeck received pseudonymized data from the insured individuals' accounts from German Pension Insurance North and calculated a risk index. This made it possible to identify a group of people who were eligible for support through AktiFAME. You were one of these individuals. Due to limited availability

Gefördert durch:

Bundesministerium für Arbeit und Soziales

aufgrund eines Beschlusses des Deutschen Bundestages

rehapro

Brücke SH

Deutsche Rentenversicherung Nord

BFW Berufsförderungswerk Stralsund

Universität zu Lübeck

during the trial period of the new service, we randomly decided who to write to and inform about the service. You have not received the offer at this time.

To test whether our new service is effective, the University of Lübeck is now surveying both groups, i.e., those people who were informed about the service and those to whom we were unable to make an offer at the time. Only if the comparison of the two groups shows an advantage in favor of the new service will we –German Pension Insurance North – transfer the service to standard care.

**What can you do?**

To evaluate the effectiveness of case management, we need your support. We can only make this evaluation if we know how you are doing today in terms of your health. Please fill out the enclosed questionnaire independently and completely. Then put it in the enclosed return envelope (fee to be paid by recipient). Then send the envelope directly to the University of Lübeck.

**Link with data from your insurance account**

By returning the letter, you agree that selected pseudonymized data from your insured person's account can be linked to the information in the questionnaire. This information relates to all rehabilitation measures applied for at the German Pension Insurance North in the last year. In addition, we – German Pension Insurance North – will inform the University of Lübeck whether you are employed.

**Voluntariness**

We would like to point out once again that completing the questionnaire is voluntary. If you decide not to complete the questionnaire, you do not need to do anything. There will be no disadvantages for you. You can also simply ignore the reminder that you will receive in about three weeks.

**Active access, counseling and case management for people at high risk of permanent work disability**

**Name of case manager**
**N. N.**

**German Pension Insurance North**
**N. N.**

Gefördert durch:

Bundesministerium für Arbeit und Soziales

rehapro

Brücke SH

Deutsche Rentenversicherung Nord

BFW Berufs-Förderungswerk Stralsund

Universität zu Lübeck

aufgrund eines Beschlusses des Deutschen Bundestages

**Request for your participation**

We would be delighted if you would complete the questionnaire. Your information is extremely valuable to us as we aim to improve and develop our services.

Of course, I am at your disposal for any further questions!

With kind regards,

N. N.

German Pension Insurance North

**Active access, counseling and case management for people at high risk of permanent work disability**

**Name of case manager**
**N. N.**

**German Pension Insurance North**
**N. N.**

Gefördert durch:

Bundesministerium für Arbeit und Soziales

aufgrund eines Beschlusses des Deutschen Bundestages

rehapro

Brücke SH

Deutsche Rentenversicherung Nord

BFW Berufsförderungswerk Stralsund

Universität zu Lübeck
