# Supplementary material for: An innovative case management intervention for people at high risk of permanent work disability to improve rehabilitation coverage and coordination of health services: a randomized controlled trial (AktiFAME, DRKS00024648)
Source: BMC Health Serv Res. 2022 Mar 15;22:342. doi: 10.1186/s12913-022-07482-9 (PMC8922787; doi:10.1186/s12913-022-07482-9)
Supplement: Supplementary file 4 — Additional file 4. Information on participation in the nested cohort study [file 12913_2022_7482_MOESM4_ESM.pdf]

INFORMATION

to participate in the scientific study accompanying

AktiFAME – Active access, counseling and case management for people at high risk of permanent work disability

Prof. Dr. Matthias Bethge
University of Lübeck
Institute for Social Medicine and Epidemiology
Ratzeburger Allee 160
23562 Lübeck

T +49 451 500 51280
F +49 451 500 51204
M matthias.bethge@uksh.de
www.aktifame.de

German Pension Insurance North, Brücke Schleswig-Holstein gGmbH and Berufsförderungswerk Stralsund GmbH have developed an innovative approach to improve the participation of people with health impairments. This approach is a case management intervention. We call it "AktiFAME." Our approach and the accompanying study are by the German Federal Ministry of Labor and Social Affairs as part of the rehapro federal funding program (https://www.modellvorhaben-rehapro.de). Prof. Dr Matthias Bethge from the Institute of Social Medicine and Epidemiology at the University of Lübeck directs the scientific study that evaluates our approach.

What questions does the scientific study clarify?

The aim of the scientific study is to clarify how many and which persons participate in the newly developed case management AktiFAME, how the new approach is implemented, which changes can be observed among the participants, and how many participants claim one or more rehabilitation measures of German Pension Insurance North after the start of the case management.

General information on data protection

In accordance with the European Data Protection Regulation (EU-DSGVO), we would like to inform you about the data we collect during the intervention and the purpose of the data we collect. In addition, we will inform you about your rights.

What data are collected?

We ask you to complete questionnaires at the beginning and end of the case management intervention and 12 months after the start of case management. You will receive the first two questionnaires from the case managers. The third questionnaire you will receive by mail. The first and third questionnaires are about your health status, your ability to work and your participation. In the second questionnaire, you have the opportunity to rate the novel case management. We receive the pseudonymized first questionnaire from the case managers. The second and third questionnaires will also be pseudonymized and sent directly from you to us (fee to be paid by the recipient). We also receive data on previous

Gefördert durch:

Bundesministerium für Arbeit und Soziales

rehapro

Brücke SH

Deutsche Rentenversicherung Nord

BFW Berufs-Förderungswerk Stralsund

Universität zu Lübeck

aufgrund eines Beschlusses des Deutschen Bundestages

employment and medical data from German Pension Insurance North. To collect these medical data, you will fill out a short form that will allow German Pension Insurance North to confirm your participation in AktiFAME. After one year, German Pension Insurance North also informs us whether you have applied for medical or vocational rehabilitation and whether you are employed. In addition, case managers document the new service (e.g. timing, duration, and type of contacts).

How do we handle the data we collect?

To protect your data, an identification number is assigned to your name. All evaluations are therefore pseudonymized, i.e. they are carried out exclusively with knowledge of your identification number, but without your name or other personal details. To ensure a transparent research process, the pseudonymized data are permanently stored in a data repository (data archive) (https://www.synapse.org/). At the University of Lübeck, all data will be completely deleted after ten years (12/31/2034). All paper documents are stored in accordance with data protection regulations on the premises of the University of Lübeck or German Insurance Pension North and are disposed of after ten years (12/31/2034) in accordance with data protection regulations.

Voluntariness of participation and your rights

Participation in the model project and in the scientific study is voluntary. If you decide not to participate, you will not suffer any disadvantages. If you wish to withdraw your participation at a later date, obtain information about your stored data, assert the restriction of the processing of your data or exercise your right to data deletion, please contact Prof. Dr Matthias Bethge at the University of Lübeck (e-mail: matthias.bethge@uksh.de). Withdrawal results in the permanent deletion of the collected data. The deletion of the data after withdrawal is possible only before complete anonymization of the data. Participation in case management without participation in the scientific study is not possible. Therefore, if participation in the study is withdrawn before the end of the case management, case management will also be terminated prematurely.

Prof. Dr. Matthias Bethge
University of Lübeck
Institute for Social Medicine and Epidemiology
Ratzeburger Allee 160
23562 Lübeck

T +49 451 500 51280
F +49 451 500 51204
M matthias.bethge@uksh.de
www.aktifame.de

Gefördert durch:

Bundesministerium für Arbeit und Soziales

aufgrund eines Beschlusses des Deutschen Bundestages

rehapro

Brücke SH

BFW Berufsförderungswerk Stralsund

Deutsche Rentenversicherung Nord

Universität zu Lübeck

In the event of a complaint, please contact: Unabhängiges Landeszentrum für Datenschutz Schleswig-Holstein
Holstenstraße 98, 24103 Kiel
E-Mail: mail@datenschutzzentrum.de

Responsible body for the research project and responsible data protection officer

Responsibility for the research project lies with Prof. Dr Matthias Bethge of the University of Lübeck (e-mail: matthias.bethge@uksh.de). If you have any questions about data protection, please contact the data protection officer at the University of Lübeck Study Centre, Dr Stefan Braun (e-mail: datenschutz@uni-luebeck.de).

If you have any questions, please feel free to contact me.

We thank you very much for your participation and support and send you our kind regards.

Matthias Bethge

Prof. Dr. Matthias Bethge
University of Lübeck
Institute for Social Medicine and Epidemiology
Ratzeburger Allee 160
23562 Lübeck

T +49 451 500 51280
F +49 451 500 51204
M matthias.bethge@uksh.de
www.aktifame.de

Gefördert durch:

Bundesministerium für Arbeit und Soziales
aufgrund eines Beschlusses des Deutschen Bundestages

rehapro

Brücke SH

BFW Berufs-Förderungswerk Stralsund

Deutsche Rentenversicherung Nord

Universität zu Lübeck
