# Supplementary material for: An innovative case management intervention for people at high risk of permanent work disability to improve rehabilitation coverage and coordination of health services: a randomized controlled trial (AktiFAME, DRKS00024648)
Source: BMC Health Serv Res. 2022 Mar 15;22:342. doi: 10.1186/s12913-022-07482-9 (PMC8922787; doi:10.1186/s12913-022-07482-9)
Supplement: Supplementary file 5 — Additional file 5. Consent form of the nested cohort study [file 12913_2022_7482_MOESM5_ESM.pdf]

AktiFAME

CONSENT

to participate in the scientific study accompanying

AktiFAME – Active access, counseling and case management for people at high risk of permanent work disability

....

First name Name

Prof. Dr. Matthias Bethge
University of Lübeck
Institute for Social Medicine and Epidemiology
Ratzeburger Allee 160
23562 Lübeck

T +49 451 500 51280
F +49 451 500 51204
M matthias.bethge@uksh.de
www.aktifame.de

I was informed about the content and the aim of the AktiFAME study. The AktiFAME study is funded by the German Federal Ministry of Labor and Social Affairs as part of the rehapro federal funding program (https://www.modellvorhaben-rehapro.de). The accompanying observational study on the implementation of case management is headed by Prof. Dr. Matthias Bethge from the University of Lübeck. The latter conducts the study and evaluates the data. A leaflet with information on data protection was given to me.

I would like to support the study through my participation and agree to complete the questionnaires that will be handed out to me. There will be no costs for me.

I agree that the data from my case manager’s documentation (e.g. time, duration and type of contacts), my questionnaire data, my medical information on participation in AktiFAME (health data), and administrative data from my pension account (employment, sociodemographic data, rehabilitation) will be merged by the researchers using an identification number. I have been assured that no personal data (name, year of birth, contact details) or other information that allows conclusions to be drawn about my person will be passed on to third parties outside the study.

Gefördert durch:

Bundesministerium für Arbeit und Soziales

aufgrund eines Beschlusses des Deutschen Bundestages

rehapro

Brücke SH

BFW Berufsförderungswerk Stralsund

Deutsche Rentenversicherung Nord

Universität zu Lübeck

I know that I can withdraw my consent at any time without giving reasons and without disadvantages. I was fully informed about my rights, but also about the time I would spend as a study participant. All my questions were answered to my satisfaction.

Under these conditions, I give my consent to participate in the study.

....	....
Place, Date	Signature

AktiFAME – Active access, counseling and case management for people at high risk of permanent work disability

Prof. Dr. Matthias Bethge
University of Lübeck
Institute for Social Medicine and Epidemiology
Ratzeburger Allee 160
23562 Lübeck

T +49 451 500 51280
F +49 451 500 51204
M matthias.bethge@uksh.de
www.aktifame.de

Gefördert durch:

Bundesministerium für Arbeit und Soziales

aufgrund eines Beschlusses des Deutschen Bundestages

rehapro

Brücke SH

BFW Berufsförderungswerk Stralsund

Deutsche Rentenversicherung Nord

Universität zu Lübeck
